# Emerging complexities and rising omission: Contrasts among socio-ecological contexts of infectious diseases, research and policy in Brazil

**DOI:** 10.1590/1678-4685-GMB-2020-0229

**Published:** 2021-03-17

**Authors:** Leandro Luiz Giatti, Ricardo Agum Ribeiro, Alessandra Ferreira Dales Nava, Jutta Gutberlet

**Affiliations:** 1 Universidade de São Paulo, Faculdade de Saúde Pública, Departamento de Saúde Ambiental, São Paulo, SP, Brazil.; 2 Instituto Federal de Rondônia (IFRO), RO, Brazil.; 3 Fiocruz Amazônia, Instituto Leônidas & Maria Deane, Laboratório de Ecologia de Doenças Transmissíveis na Amazônia, Manaus, AM, Brazil.; 4 University of Victoria, Department of Geography, Victoria, BC, Canada.

**Keywords:** Amazon, emerging diseases, Anthropocene, environmental changes, public policy

## Abstract

In this article, we explore elements that highlight the interdependent nature of demands for knowledge production and decision-making related to the appearance of emerging diseases. To this end, we refer to scientific production and current contextual evidence to verify situations mainly related to the Brazilian Amazon, which suffers systematic disturbances and is characterized as a possible source of pathogenic microorganisms. With the acceleration of the Anthropocene's environmental changes, socio-ecological instabilities and the possibility of the emergence of infectious diseases merge into a background of a ´twin insurgency´. Furthermore, there is a tendency to impose economic hegemony in the current Brazilian context, corroborating discourses and pressures to a scientific simplification and denial. With this, we assert that developmental sectoral actions and monoculture of knowledge characterize an agenda of omission, that is, a process of decision making that indirectly reinforces ecological degradation and carelessness in the face of the possibility of the emergence and spreading of new diseases, such as COVID-19. Tackling the socio-ecological complexity inherent in the risk of the emergence of infectious diseases requires robust co-construction of scientific knowledge, eco-social approaches, and corresponding governance and sophisticated decision-making arrangements.

## Introduction

The emergence of the COVID-19 pandemic brutally interferes with all segments and dynamics of human activities in the critical current context. Inevitably, this crisis of unprecedented dimensions reveals processes of interdependence, vulnerabilities, uncertainties and interactions that are of great concern to various critical and challenging situations for all humankind. Thus, the effects and threats of this and other emerging and re-emerging epidemics prompt analyses and demand dialogues about climate change, political instability, socio-environmental vulnerability, global scarcity of environmental resources, destruction of ecosystems, migrations, and unequal effects on marginalized social groups.

Faced with this high complexity, we emphasize the need to explore and discuss two relevant axes of problematization in relation to the Brazilian context, which inevitably interact globally regarding vulnerability in the face of any emerging disease as impactful as COVID-19. The first axis is characterized by the need to value the interdisciplinary knowledge required to understand the emergency and to direct actions that can be effective. For that, plural knowledge, multisectoral actions and the involvement of different social actors must be strengthened. Such reinforcements are needed to mitigate the effects of the current pandemic, as well as to prevent other emerging diseases with similar threats (Parkes *et al*., 2005; Hancock, 2015; Hotez, 2016). The second axis is the need for critical reflection and control actions regarding the resumption of a predatory development model that is highly aggressive to ecosystems, capable of exacerbating risks of re-emergence or emergence of diseases. Thus, it is imperative to consider the Amazon and other Brazilian biomes, which are suffering an intensification of impacts due to deforestation, forest fires, mining, extensive single-crop agriculture, cattle raising, biodiversity loss, changing of human ecological interactions, and hunting for bushmeat consumption; since the related ecosystems are biologically diverse and possibly also reservoirs of innumerable pathogens at risk of spilling over to human populations (Daszak *et al*., 2001; Patz *et al*., 2004; Wolfe *et al*., 2005; van Vliet *et al*., 2014; Nava *et al*., 2017; Cyranoski, 2020; Ellwanger *et al*., 2020). Among the drivers for these impacts are illicit activities, instabilities, violence, corruption, and the emptying of the state apparatus. These factors are responsible for the increased risk of new and old diseases spreading from ecosystems, successfully disseminating among humans (Hirschfeld, 2020).

In this article, we explore some elements that highlight the interdependent nature of the demands for knowledge production and decision making in situations that characterize the emergence of infectious diseases. In this regard, we focus on Brazilian contexts such as those inherent to the Amazon region. We also dialogue with the current political scenario based on discourses and decision-making towards the production of knowledge to confront the COVID-19 crisis to conserve (or not conserve) the country's natural heritage. Hence, we start with a non-systematized bibliographic review that is guided by recognized and current academic production on intersecting themes. In addition, we also use information from media publications (primarily with references, investigative journalism, and interviews with specialists), mainly to inform the current Brazilian context through a factual basis, given the immediate nature of the situations discussed. We identify media information by citation in the text, referenced in the Internet Resources Section.

## Emerging diseases in the social-ecological context of the Anthropocene

Despite the persistent scientific challenge and some conspiracy theories, there is strong evidence that the new coronavirus (SARS-CoV-2), which emerged in Wuhan in China, has its origin in wild animals. Malayan pangolins (*Manis javanica*) were identified as possible intermediate hosts for SARS-CoV-2 that may transmit the coronavirus for humans; Recently sequence-based analysis suggested bats as a key reservoir for SARS-CoV-2, accordingly with SARS phylogenetic tree (Lam *et al*., 2020; Shereen *et al*., 2020; Zhang *et al*., 2020). The point is that the disease originated in a natural reservoir and its process of mutation and human infection is related to interactions and anthropic impacts on ecosystems and their biological communities (Cyranoski, 2020; Zhou *et al*., 2020).

Several infectious viral diseases have already been understood by their origins from natural reservoirs. In fact, situations with emerging new diseases indicate that various determining factors are combined with environmental impacts, such as changes in socio-ecological dynamics and respective social injustices and vulnerabilities, as we observe in the example of the rise of the Nipah virus in Malaysia, in 1998. This zoonosis, caused by a highly virulent paramyxovirus, has as its natural reservoir species of fruit bats and was transmitted to humans through pigs as intermediate hosts. Global pressures from expanding pig farming and diverse impacts on ecosystems have contributed to the process of disease dissemination in that country. An ecological hypothesis for the emergence of this disease considers that deforestation for agricultural expansion has caused bats to look for fruits on farms, including pig farms (Parkes *et al*., 2005). Another example is the international emergence of Ebola in West Africa, characterized as an unprecedented situation in terms of scale and impact between 2014 and 2015. Ebola is also a viral zoonosis that is highly virulent to humans and whose infection comes from natural reservoirs of primates and bats. Socio-ecological dynamics can mediate its emergence and spread, in correlation with processes of ecosystem degradation, lack of healthcare and health surveillance, instabilities and armed conflicts, all aggravating the epidemic (Pigott *et al*., 2014; Heymann *et al*., 2015).

With regard to the Brazilian Amazon, a vast history of developmental actions and several anthropogenic actions of impacts to ecosystems allow us to understand relationships between landscape transitions and the dissemination or prevalence of infectious diseases (Confalonieri, 2005). Moreover, global environmental changes like climate change combined with biodiversity loss in dynamic socio-ecological disturbances (deforestation, migrations and the advance of the agricultural frontier, large infrastructure projects such as hydroelectric dams, roads and the construction of railways, mining and urbanization) have favored a wide range of consequences in the proliferation of numerous infectious diseases in the Amazon region (Ellwanger *et al*., 2020).

Land-use change is one of the main triggers for emerging zoonotic diseases (Jones *et al*., 2008). The conversion of a forested area into pasture, soybean or sugarcane plantation and dams construction can cause zoonotic agents to spill over and represents a serious threat to the health of local communities. This process reduces biodiversity and alters ecological patterns and favors taxonomic groups, such as small mammals, particularly rodents and bats that are less sensitive to disturbances (Loh *et al*., 2016) and competent reservoirs of many known zoonotic diseases. Land-use change also can alter the types of human-wildlife contact, increasing the chances for outbreaks and selection of pathogens´ mutation events and disruption of the community ecology of infectious agents by decreasing species richness (Murray and Daszak, 2013; Loh *et al*., 2016; Nava *et al*., 2017).

Increased devastation of the Brazilian Amazon is a likely tragedy due to the impact on uncountable essential ecosystem services this ecosystem provides, like carbon storage in soils and biomass, modulation of regional climate patterns, and regulation of water and nutrients cycles, regional water balance and river flows. The ecosystem services of the Amazon are very important for agriculture in the region itself, but also outside its domains, mainly because of its influence through atmospheric circulation and precipitation as a continuous part of Earth system operations (Boerner *et al*., 2007; Foley *et al*., 2007; Malhi *et al*., 2008). However, the tragic consequences can also be the imminent risk of emerging zoonotic infectious diseases outbreaks, such as SARS-CoV-2. The Amazon rainforest, as other high biodiversity ecosystems is host of numerous and unknown viruses (Jones *et al*., 2008). Several studies found that reducing anthropogenic activity as land-use change, and conserving areas with high wildlife diversity also can reduce the chances of zoonotic disease emergence (Jones *et al*., 2008; Ostfeld, 2009). Protection of high biodiversity ecosystems should become a public policy related to public health, in order to protect humans against epidemics originated from zoonotic sources (Ostfeld, 2009; Nava *et al*., 2017).

Recently, a podcast contemplated a relevant historical episode in dialogue with the current pandemic. It relates to the plague of Athens that is estimated by killing up to half of Greece’s urban population almost 2,500 years ago - the cause of the plague of Athens is a constant debate among experts, the symptoms and the mode of spread indicate that it was an infectious disease, but more recently, some authors suppose that the disease was typhoid fever (Papagrigorakis *et al*., 2006). While important learnings, failures and disorders of society allow for the success of the etiological agent, which condemns a civilization to the worst outcomes. In analogy of this historical episode with the current context of COVID-19, university professors - from the political science and classical studies areas -, argue on two divergent paths: organization, leadership, concise public policies and stability may lead to scenarios equivalent to the contexts of New Zealand or South Korea; the opposite may lead to profound consequences, such as those verified in the United States and Brazil (CBC/Radio-Canada, 2020).

However, a critical point that needs to be addressed is that in the historical episodes such as the plague of Athens or the Spanish flu, the emerging process of these diseases is structurally different from the zoonotic disease pandemic and outbreaks nowadays. For the current health challenges, the zoonotic source can be differently disrupting (or modulating) by our actions towards the environment. It is because since the 1950s anthropogenic impacts on the biosphere increased very dramatically and became globally interconnected through scarcity and the depletion of resources. On the other hand, such acceleration characterized rapid rates of urbanization and huge population growth, massive vulnerabilities and very dynamic connectivity, furthered by international travels, all of this adding to exacerbate the perspective of systemic emergencies (Steffen *et al*., 2015).

The recognition of the magnitude of these human-dominated contexts characterizes the denomination of a new geological epoch, the Anthropocene, in which humanity places itself as a force of change of planetary-scale (Crutzen, 2002). The consequent natural resources exhaustion, biological extinction, and climate change indicate that the world seems to have become smaller, closer and ruled by the phenomenology of interdependent scarcity and risks.

As we can see in the severe and comprehensive crisis of COVID-19 and its far-reaching consequences on social and economic dynamics, a pandemic can also be understood within the spectrum of the Anthropocene transformations (Heyd, 2020). After intense struggles and advances in the 20th century in search of social inclusion, sustainability, justice, public health, scientific advances and certainties to reduce and control infectious diseases, the contemporary brings us somewhat unexpected surprises. At the turn of the 21st century, crises and setbacks are combined with the exacerbation of the global environmental crisis of unsustainability and the development of side effects such as epidemics. Thus, we have political instabilities, wars and different armed conflicts, abusive commercial practices, reduction of the role of the state as a provider for human development, failures in the necessary collaboration and in global governance, environmental injustices associated with environmental degradation and criminal conduct, imprudence regarding the limits of ecosystems, and overpopulation, rapid urbanization and intensification of human international mobility. Regarding these combined circumstances, on the one hand, we have a microbial insurgency characterized as a symptom of ecological imbalances expressed in the emergence of new infectious diseases. On the other hand, we have a series of intertwined factors, insufficiencies and instabilities that contribute to the wide spread of these diseases in catastrophic proportions. Both entail a complex process of 'twin insurgency', that can corroborate with a regression in the concept of epidemiological transition, due to the conjuncture of new and overwhelming epidemics by infectious diseases (Hirschfeld, 2020).

This all demonstrates that we are confronted with complex, persistent and exacerbated challenges that are not easy to solve and require learning processes that also involve different social actors. The expert and academic mainstream knowledge dedicated to solution-oriented approaches to problems can be considered insufficient in the face of the current complexities and uncertainties. The complexity of present-day society requires alternative approaches to societal and environmental challenges, which means to listen to multiple voices and institutions, and involve a plurality of stakeholders in the decision-making (Sardar, 2010; Giatti, 2019; Scoones and Stirling, 2020).

## The current Brazilian and Amazonian context for science and policy

The discussion established here aims at providing an overview of policies focused on the Amazon, as well as pointing towards the narrowing current stance of the federal government regarding science. This shows that such visions can be understood as complementary, especially if analyzed in relation to mistaken policies and based on an agenda characterized by monoculture of knowledge (Santos, 2007; Santos *et al*., 2016), contrasting with the multiple socio-cultural and ecological dimensions of causality.

The Brazilian Amazon region is historically marked by dichotomous public policies of economic and social development. In order to exemplify the Amazonian context, concerning public policy projects for social and economic development, we will have an arrangement of actions and we can observe that such practices interfere structurally in their organizational manner, resulting in greater disorganization in the public health crisis faced by COVID-19, in this region.

First, the existence of the original indigenous peoples occupying the land has been historically ignored by the Brazilian rulers, who promote the development and settlement of the Amazon region through the implementation of governmental actions focused on unilateral policies. The region is deeply marked by cycles of resource exploitation for rubber, wood, soybean, minerals and livestock. Similar to demystifying the false idea of Terra nullius, applied to many other colonies with the purpose to rip the people off their land and to facilitate occupation, in ´*The Invention of the Amazon*´ (Gondim, 2007), we learn about the fact that the Amazon was not discovered and that instead there was an ethnocentric invention on the territory and its people (a different term should be applied instead of discovery since the history of Amazon and Brazil did not begin with the arrival of the Europeans, what should emphasize the presence of native peoples in Brazilian historiography). Since the 1940s, the Amazon region has become the focus of a government strategy of occupation and intervention. Since then, forest exploitation policies were intensified as a measure of regional development.

In Brazil's economic formation, the economist Celso Furtado builds an economic classification for the country based on economic cycles (Furtado, 2007). For that matter, it would not be an exaggeration to assume that the Amazon region, although mistakenly, also surrenders to such an economic development pattern. Accordingly, we can list the rubber cycles (late 19th century and the 1930/40s); and those cycles that started in the 1960s: wood, soybean, minerals and cattle raising. These cycles were strongly encouraged and enforced during the period of Brazil's military regime (1964 - 1985).

These policies were intended to occupy and economically develop the region, but not to understand, study and contribute to human development and ecological conservation (Giaretta *et al*., 2019; Silva and Lucas, 2019). The policies implemented were top-down (Sabatier, 1986), reverberating in close discrepancy between their application and practice. In the context of COVID -19, current policy evolves as a complete disarticulation of what actually is needed to formulate and implement distinct and inclusive, indigenous policies, necessary for this multifaceted region. In addition, the Brazilian institutional design, decentralized as to the application of the actions, is also extremely dependent on the central (federal) power as organizing and financing actions.

We can draw a brief analogy between the ordering of public policies established in the North of Brazil (Amazon) in comparison with the South/Southeast regions in the country. These policies show evidence that in the face of a long and deep history of submission to national desires, the North of Brazil finds itself in a disadvantageous situation during the fight against COVID-19. The political and also economic choices made throughout decades have regarded the Brazilian Amazon as a negative place, which consequently hampers the capacity to respond to the problems arising from the public health crisis. That is a context created by centuries of omission in terms of public policies directed towards the local populations.

Since the colonial period, the political focus for the Amazon was on exploratory and predatory practices, without responsibly considering the local population or even the settlers that came to ´occupy´ the region. As a result of these exploitive longstanding politics, the Amazon today is deeply marked by appropriation and dismantling. These aspects are becoming more evident with the arrival of the COVID-19 pandemic in 2020, translating into severe inequities and unjustices. As so, the numbers from the Ministry of Health (June 2020) pointed to 13 times more deaths proportionally, due to the coronavirus, in the North of Brazil compared to the South (Folha de S. Paulo, 2020a).

In July 2020 Brazil had a total of 13,728 cases of COVID 19 among indigenous population with 1.716 incidence and lethality of 1.86% (Simionatto *et al*., 2020). The last SESAI (Subsistema de atenção à saúde indígena) report (10/19/2020) describes a total of 31,327 cases of COVID 19 among Brazilian indigenous population with 464 deaths (Saúde Indígena, 2020). Amazon concentrates most of the Brazilian indigenous population, which today is of 896,917 with 57.7% living on indigenous lands in situations of vulnerability and insufficiency of health care regarding the current risk of COVID-19 dissemination. In this context, there has been an increase of deaths due to respiratory failure in 2020 among Brazilian indigenous, possibly caused by COVID-19, but not confirmed (Palamim *et al*., 2020).

A recent debate about uncertainties led by Edgard Morin on COVID-19 (Instituto Humanitas Unisinos, 2020) finds an echo when we analyze the choices that determined public policies in the Amazon. These policies carry a great trend of causality supported by reductionist thoughts, based on non-recognition of ecological and social diversity. This prevents more complex initiatives for problem perception and resolution. We can assume that, given the conventional political and economic scenario, the uncertainties in the North of the country, are in fact the prevailing certainties.

At a general level, we identify that conflicting situations and instabilities have permeated the current Brazilian scenario with respect to the confrontation of COVID-19 and the conduction of policies for the preservation of natural resources, especially in the Amazon. In this sense, a particular concern lies in government discourses and positions that confront scientific production, especially counting on the misfortunes of public research institutions with a high international reputation.

The currently prevailing option of the Brazilian federal government in addressing the impacts of the pandemic is to ensure the conditions for regular economic activities. Even when reaching the figure of 150,000 deaths by COVID-19 (10/10/2020), the president of Brazil, Jair Bolsonaro, continued to minimize the pandemic and to speak out demanding state governors to reopen trade and relax preventive measures to control the virus dissemination (UOL, 2020a). Similarly to the United States (El País, 2020a), the Brazilian federal government also antagonized the positions of some state governors who acted with more prudence following sanitary and scientific recommendations.

One of the most prominent issues in the postures of the federal government was the constant bet on the use of chloroquine and hydroxychloroquine in patients with COVID-19, even though with no substantial proof of the drug's effectiveness. This posture corroborates as one of the possible disagreements that caused the resignation of the second minister of health to leave the government during the pandemic. After this episode occurred in May 2020, the army general Eduardo Pazuello became the interim in the ministry of health (UOL, 2020b), and turned effective in the position in September 2020. The presence of the general, who has no training or experience in the area of health (BBC, 2020), composes a set of evidences of an intervention process in the ministry of health, in which military personnel is occupying key positions without having the appropriate qualification. All these facts explain why it has been difficult to have a national coordination dealing with the pandemic within Brazil´s Unified Public Health System (SUS) (El País, 2020b). SUS could be a very relevant system in terms of controlling and preventing infectious diseases, as being public and dedicated to universal health coverage for the Brazilian population. SUS is integrated through federal, state and municipal actions, promoting primary health care, and having great capillarity especially in more vulnerable communities. 

The insistence of the Brazilian federal government on the use of chloroquine to treat the virus infection, has created a constant clash with science (G1, 2020a). The National Council of Health Secretariats (CONASS) has taken a severe stand, questioning the imposition of a chloroquine use protocol without sufficient scientific evidence (ABRASCO, 2020). On the other hand, the digital network of Bolsonaro´s supporters promoted virtual attacks and threats to researchers who, for example, released preliminary results challenging the efficiency of chloroquine (Correio Braziliense, 2020).

The environmental issue has also been the target of constant antagonism in the positions of the current Brazilian federal government, showing outdated visions on the environmental issue, some enforced during the military regime. For instance, during the COVID-19 pandemic, huge political pressure is moving to accelerate Amazon forest destruction by different drivers, mainly land-use change for agriculture and cattle ranching. In a government internal meeting, the Brazilian Ministry of Environment, Ricardo Salles, stated that the pandemic is an opportunity to weaken the environmental regulations, as people and media "just talk about COVID" (G1, 2020b). At the beginning of Bolsonaro´s government, in 2019, prior to the pandemic, in the face of a large increase in deforestation and the number of forest fires in the Amazon, the president had already refused relevant environmental information produced by the National Institute for Space Research (INPE), including exonerating the director of this international renown public institution (Folha de S. Paulo, 2020b). The conduct of the federal government and its Ministry of Environment has given evidence of a scrapping and replacement of environmental protection institutions through militarization (El País, 2020b; UOL, 2020c). All of this demonstrates the current federal government's remarkable contempt for the environmental issue and the preservation of the Amazon (Deutsche Welle, 2020).

In this context, public research and environmental protection institutions have been confronted, as well as public universities that have been targets of slander and resource cuts. The former Minister of Education, Abraham Weintraub, openly declared, a week before his resignation (06/19/2020), that he did not want his tax money to be used to train students in sociology, anthropology and philosophy (UOL, 2020d), a blatant ignorance of sharing experiences and disciplines for a multifaceted understanding of Brazilian reality. This same protagonist, in November 2019, had accused federal public universities, without evidence, of running extensive cannabis plantations and chemistry laboratories dedicated to the production of methamphetamine (G1, 2020c). In addition, in April 2020, a federal call for scientific initiation scholarships dedicated to undergraduate students excluded the human sciences (Folha de S. Paulo, 2020c) and this was indicative of the federal government's aversion to certain areas of knowledge.

The brief factual and political discourse overview provided by these events and current conjunctures in Brazil places the basis for important discussions within the scope of the objectives of this article. First, we denote a very serious context of denial to science or of selective and extremely restricted use of technical and scientific knowledge. Second, we observe serious difficulties for hybrid and interdisciplinary forms of knowledge to be favored in the current national context. That is, the opposite sense of what we should expect in terms of the knowledge that is appropriate to the inherent complexity of the emergence and spread of diseases, such as COVID-19. Thirdly, the current conjuncture signals a growing threat to ecosystems, especially with regard to the deforestation of the Amazon, the impacts on indigenous communities and the weakening of institutions responsible for their protection. This demonstrates the prevalence of predatory economic discourses in relation to natural resources, but which refutes the intrinsic importance of ecosystems and their services and biodiversity (Kulevicz *et al*., 2020).

Regarding the re-production and application of this colonial monoculture of knowledge, economic rationality is imposed on winning. Thus, it conditions a cognitive subtraction about the magnitude of the disease and the complexity of the effects of the pandemic on society and, also, on the economy itself. This hegemony is shaped by the way in which academic areas of critical reflections, such as the human sciences, are fought and oppressed. Likewise, the widely produced knowledge on the intrinsic value of the Amazon, on its ecosystem services, biodiversity and consequences of its degradation, such as the possibility of the emergence of new pathogenic agents, are also ignored. There are several examples of this one-sidedness of developmental and extractive processes with the occurrence of diseases associated with ecosystems in the recent history of the Amazon. Only with respect to the construction of the BR-230 highway in the 1970s, intense population movement and environmental impacts were associated with the spread of leptospirosis, leishmaniasis, Chagas disease, bacterial infections, malaria, Mayaro fever, and yellow fewer. Also, mining activities can be related to the occurrence of hantavirus pulmonary syndrome, and new human settlements associated with deforestation can cause changes in arthropods and bats hematophagy patterns, spreading rabies and other diseases that were previously under epizootiological equilibrium in non-human primate populations, for instance (Ellwanger *et al*., 2020).

This rude and reductionist rationale forms the basis for simplifying decision-making models, taking unilateral decisions and other inconsequential ways of dialoguing with problems that cannot be dealt separately. In the field of political science studies, there is a definition of public policy that consists of all that a government decides to do or not to do (Dye, 1992). Therefore, when it comes to unilateral economic desicion-making, ignoring ecology, public health and social context, this is not only something that does not fit on the agenda. Indeed, this is a political decision, a resolution to channel actions that oppress other knowledges and ignore wider phenomenologies. In this sense, the ways of minimizing the pandemic, despising the environment, discrediting science and militarizing technical-scientific areas characterize a process of omission that is reflected in the design of an ongoing, colonial governmental policy. Therefore, there is no ignorance and lack of sensitivity, what certainly exists is a process of decision and action.

## Eco-social approaches and new governance on complex phenomena

The problems we are facing today - be it climate change, species extinction, or the spread of deadly viruses - are complex, intertwined, and are present at several system levels; and different societal actors are involved. Often there is neither agreement about the definition of the specific challenges nor about the most adequate solution, thus uncertainty is not the exception but the rule. New approaches to knowledge creation are needed in order to resolve these issues. Wenger (1998) has called ‘Community of Practice’ the mutual involvement of participants, a horizontal and shared, decolonial process of learning, determined by all participants together, using all available skills, assets and resources which are involved and developed over time to create knowledge; which involves culture, language, instruments and technology, routines, ways of performing, stories, symbols and gestures. Local knowledge becomes part of this alternative epistemology, where through shared practices new knowledge and innovations are created. The complexity of current social and environmental challenges demands these new ways of knowledge production, where local and scientific knowledges are integrated to address problems and where local communities are engaged in the responsibilities of sustainable resource management (Giatti, 2019). The United Nations 2030 Agenda is focused on Sustainable Development to address the actual dynamics of this ongoing health crisis; and while there is a link relating human pressures leading to environmental degradation, the link between environmental change and human health impact is usually not integrated in sustainable development planning (Di Marco *et al*., 2020).

The serious current crisis initiated by a pandemic has unleashed numerous other emergencies and tragedies. Covid-19 has exposed the vulnerability of humans, and our interdependency with other living beings and with the environment and climate. However, the pandemic has also uncovered unlimited creativity and resilience in the human mind and spirit. The word crisis derives from the Greek word “decision”, meaning a time of intense difficulties, problems or danger, a time when a challenging or important decision must be made.

A crisis, as tragic as it is, always offers opportunities. We must not waste this chance and we must demand profound political changes to correct the social and environmental injustices that affect the lives of large quantities of people and the environment as a whole. In this regard, the historical particularities and tendencies of policies towards the Amazon and the Brazilian context itself motivate us to call for more integrated, participatory and democratic solutions and knowledge. That is what Ventura *et al*. (2020) stress on the need for a permanent research and surveillance agenda that not only considers the evolution of specific diseases but encompasses intrinsic factors from social, environmental, economic and political determinants of epidemics. This must be associated with international cooperation and dialog capturing local dynamics within eco-social integrated approaches (Parkes *et al*., 2005; Hancock, 2015), strengthening interdisciplinary knowledge for the organization of plural governance structures appropriate in addressing human health challenges in the Anthropocene (Whitmee *et al*., 2015).

**Figure 1 -:**
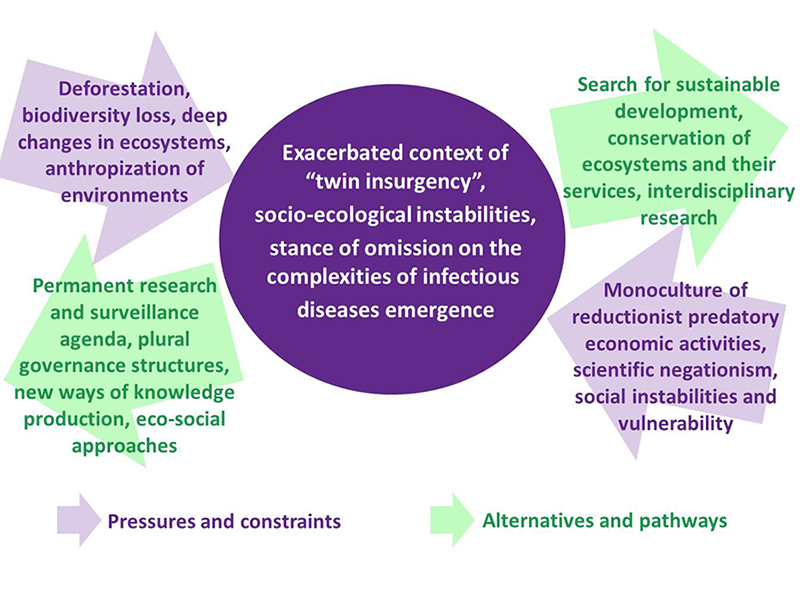
Model of pressures and alternatives to the context of twin insurgency of emergent infectious diseases.

However, the current Brazilian scenario is very critical and averse to what we understand as the demanded approaches. Although the very issue of interdisciplinarity places the country as a leader because of its scientific production (van Noorden, 2015), the context of the federal government's current guidelines imposes itself as a setback in this sense. In addition, the extreme right-wing political context and its way of confronting scientific production (Jacques *et al*., 2008; Hansson, 2017) is added to the rescue of outdated paradigms on the exploitation of natural resources. All this characterizes the two paths identified by Hirschfeld (2020) as propitiating a 'twin insurgency', conforming to the worrying situation of disease emergence in the scope of wide instabilities capable of deep exacerbation of consequences. The picture leads us to reflect on Brazil and the world's current risks, considering the spread of COVID-19 or the emergence of other new diseases. Considering the Brazilian Amazon context and the current political scenario we present Figure 1 exposing the main factors that contribute to a twin insurgency of emergent infectious diseases in the face of instabilities, monoculture of knowledge and the consequent one-sidedness of developmental processes. Also, some remarkable alternatives and pathways are presented to mitigate such a trend.

We are at a turning point when major change can occur, either towards recovery or towards fatality. This brings to the forefront the questioning and examining of the nature and the effectiveness of our current governance systems, understood as defining formal and informal rules, distributing roles, outlining practices, creating and setting boundaries, reflecting and deciding in consideration of uncertainties, and influencing behaviors for the purpose of achieving desirable and sustainable collective outcomes (Basel Institute on Governance, 2020).

We are urged to address the complexities and challenges our societies are facing, recognizing and respecting the local realities and customs. We should do much more to prepare for the global, collective challenges that await us in the future, particularly related to climate change, mass extinction, public health and poverty. This crisis allows us to transition into a more sustainable future, by assessing the strengths and weaknesses of different models of governance, moving beyond the state and towards the empowerment of the people, respecting democratic rights and freedoms and making participatory democracy an everyday practice and not just a theoretical concept. An interactive environmental governance perspective with “*interventions aiming at changes in environment-related incentives, knowledge, institutions, decision making, and behaviors*” is imperative now (Lemos and Agrawal, 2006).

Building interdisciplinarity and governance systems that are participatory and effective is on the global agenda. Most of all, however, we recognize that this is a learning process, where different knowledges contribute to raising consciousness, creating understanding and finding solutions to major complex challenges. More democratic approaches and plural knowledge must be encouraged to break with policies of omission and exclusion, based on the monoculture of knowledge. Only such approaches and perspectives can be robust enough to cope with complex situations related to emerging diseases in the contemporary context. In such a proposed frame, there must be social mobilization and plurality against predatory, colonial, oppressive and reductionist projects.
